# Quantitative proteomic characterization of lung-MSC and bone marrow-MSC using DIA-mass spectrometry

**DOI:** 10.1038/s41598-017-09127-y

**Published:** 2017-08-24

**Authors:** Sara Rolandsson Enes, Emma Åhrman, Anitha Palani, Oskar Hallgren, Leif Bjermer, Anders Malmström, Stefan Scheding, Johan Malmström, Gunilla Westergren-Thorsson

**Affiliations:** 1 Department of Experimental Medical Science, Lung Biology Unit, Lund University, 22184 Lund, Sweden; 2 Department of Clinical Sciences Lund, Division of Infection Medicine, Lund University, 22184 Lund, Sweden; 3 Department of Experimental Medical Science, Matrix Biology, Lund University, 22184 Lund, Sweden; 4 Department of Respiratory Medicine and Allergology, Lund University and Skåne University Hospital, 22184 Lund, Sweden; 5 Lund Stem Cell Center, Lund University, 22184 Lund, Sweden; 6 Department of Hematology, Skåne University Hospital, 22184 Lund, Sweden

## Abstract

Mesenchymal stromal cells (MSC) are ideal candidates for cell therapies, due to their immune-regulatory and regenerative properties. We have previously reported that lung-derived MSC are tissue-resident cells with lung-specific properties compared to bone marrow-derived MSC. Assessing relevant molecular differences between lung-MSC and bone marrow-MSC is important, given that such differences may impact their behavior and potential therapeutic use. Here, we present an in-depth mass spectrometry (MS) based strategy to investigate the proteomes of lung-MSC and bone marrow-MSC. The MS-strategy relies on label free quantitative data-independent acquisition (DIA) analysis and targeted data analysis using a MSC specific spectral library. We identified several significantly differentially expressed proteins between lung-MSC and bone marrow-MSC within the cell layer (352 proteins) and in the conditioned medium (49 proteins). Bioinformatics analysis revealed differences in regulation of cell proliferation, which was functionally confirmed by decreasing proliferation rate through Cytochrome P450 stimulation. Our study reveals important differences within proteome and matrisome profiles between lung- and bone marrow-derived MSC that may influence their behavior and affect the clinical outcome when used for cell-therapy.

## Introduction

Cell therapy has been under active development for the treatment of a wide array of lung disorders. In particular, mesenchymal stromal cells have been given intense attention due to their low or absent HLA class II expression as well as their immune-regulatory and regenerative properties. MSC isolated from bone marrow aspirates are frequently used in pre-clinical studies and clinical trials^1, 2^. However, we have recently reported that tissue-resident lung-derived MSC possess lung-specific properties compared to the bone marrow-derived MSC, such as lacking *in vivo* bone formation capacity, secretion of different cytokines, increased colony-forming capacity, and proliferation rate, all of which might effect the clinical outcome^3, 4^. Therefore, it is an urgent and important need for a detailed characterization of the molecular differences underlying these phenotypes, *i*.*e*. lung-MSC and bone marrow-MSC, in order to understand how these differences may impact cell behavior and potential therapeutic use. Importantly, a driver of cell behavior is the extracellular environment (ECM) surrounding the cells, which is regulated by the mechanical properties and the composition of the matrix^5^. The ECM molecules are mainly produced locally by fibroblasts and by their activated equivalent, the myofibroblasts. However, it is also known that other cell types to some extent can produce ECM molecules^6^. Recent advances in mass spectrometry (MS) based proteomics have made it possible to perform quantitative analysis of the MSC proteome using data-independent acquisition (DIA) MS, and improved bioinformatics analysis, such as the *in silico* definition of ECM, allow to characterize the ECM molecules produced by MSC isolated from different origins. The *in silico* definition of ECM molecules, referred as the matrisome was developed by Naba *et al*.. The matrisome list is divided in to two predominant groups, the core matrisome and the matrisome-associated proteins. Where the core matrisome consists of structural proteins such as collagens, laminins and proteoglycans. The matrisome-associated proteins on the other hand, consists of growth factors, enzymes and cytokines with ability to modify the ECM^7^.

In this study, we have performed an in depth quantitative characterization of the proteome and the matrisome profiles of both, cell layer and conditioned medium, MSC isolated from lung tissue and bone marrow aspirates utilizing DIA-MS. Our results show that the proteomes of MSC from lung tissue and bone marrow contain cell-specific proteins that influence their behavior. This tissue-specificity was also demonstrated in the ECM profile of MSC. As a proof of principle we were able to demonstrate that increasing the expression of CYP1B1 in lung-MSC decreased MSC proliferation rates.

## Results

### Lung-derived and bone marrow-derived MSC display a typical MSC surface marker profile

**Figure 1:**
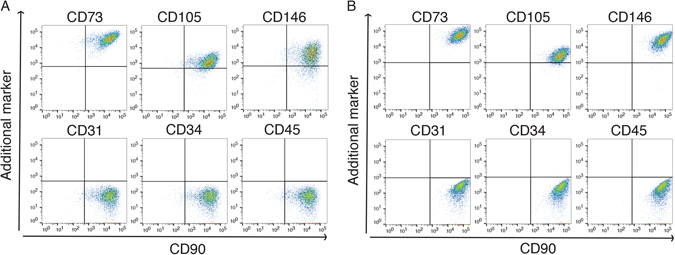
Lung-derived and bone marrow-derived MSC show typical MSC surface marker profiles. Flow cytometry analysis of cultured (passage 3–4) MSC isolated from (**A**) lung tissue (n = 3) and (**B**) bone marrow aspirates (n = 3). The x-axis indicates CD90 expression and the y-axis represents the expression of the additional marker indicated on top of the plots. Cells were positive for the surface markers CD73, CD90, CD105, and CD146, but negative for the surface markers CD31, CD34, and CD45. Data are presented as dot plots. Gates were set according to corresponding isotype control.

Immunophenotyping of lung-derived and bone marrow-derived MSC using multicolor flow cytometry demonstrated a typical MSC surface marker profile. Lung- and bone marrow-derived MSC were positive for CD73, CD90, CD105, and CD146, but lack the expression of CD31, CD34, and CD45 (Fig. 1). In accordance with what we have published previously^3^, no differences in surface marker expression between lung- and bone marrow-derived MSC were observed (Fig. 1).

### In-depth label-free quantitative proteomic analysis of lung- and bone marrow-derived MSC

**Figure 2:**
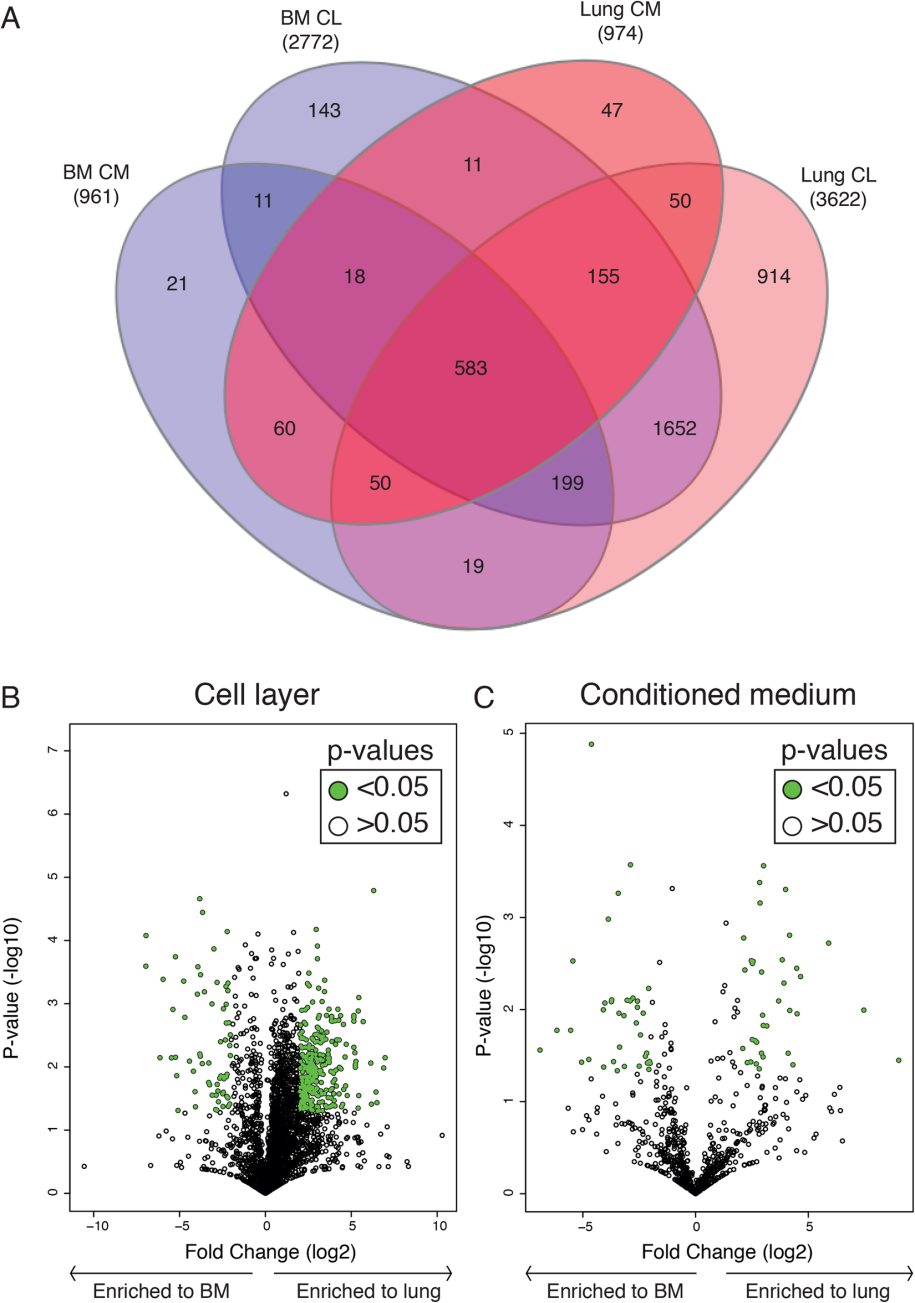
Label-free quantitative characterization of lung- and bone marrow-derived MSC proteome profiles using DIA-MS. (**A**) Venn diagram of the total number of proteins identified by quantitative proteomics in each sample group (BM-MSC conditioned medium (n = 3), BM-MSC cell layer (n = 3), lung-MSC conditioned medium (n = 3) and lung-MSC cell layer (n = 3)). Volcano plots for cell layer (**B**) and conditioned medium (**C**) for lung-MSC vs. bone marrow-MSC. Green dots indicate proteins that are significantly differentially expressed and have a fold change of ≥4 or ≤−4 and a p-value ≤ 0.05. The p-values were Benjamini and Hochberg corrected for multiple testing and presented as −log10. BM, bone marrow-derived MSC; Lung, lung-derived MSC; CL, cell layer; CM, conditioned medium.

Lung- and bone marrow-derived MSC were cultured for 24 hours in serum free medium. The conditioned medium and cell layer were collected and analyzed using label-free DIA-MS. A complete list of identified proteins is available on PeptideAtlas. Briefly, analyzing the conditioned medium collected from lung-derived MSC resulted in a total identification of 974 proteins, compared to the bone marrow-derived MSC containing 961 proteins (Fig. 2a). Analysis of the lung-derived cell layer resulted in 3622 identified proteins, while 2772 proteins were detected in the cell layer produced by the bone marrow-derived MSC (Fig. 2a). As an internal control of the proteomic analysis, we confirmed the existence of the proteins required to meet the minimal criteria for MSC classification^8^ CD73, CD90, and CD105 across all samples (Supplementary Table 1). Importantly, the proteins CD31, CD34, and CD45 were not found (Supplementary Table 1).

### Profound differences in the proteome of lung- and bone marrow-derived MSC

**Figure 3:**
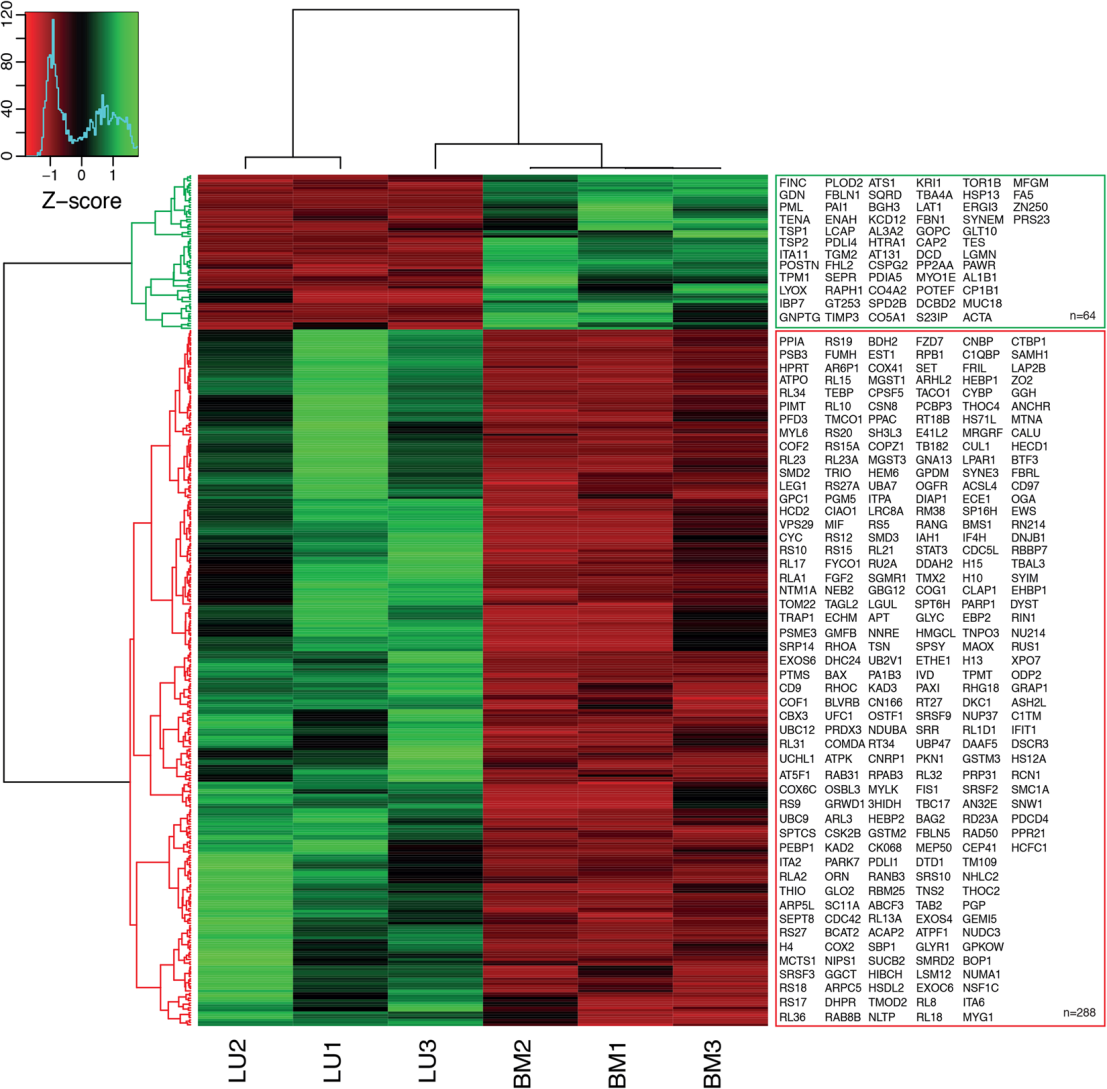
Clustering of significantly differentially expressed proteins isolated from cell layer produced by lung- and bone marrow-derived MSC. A heat map of differentially expressed proteins with a fold change of ≥4 or ≤−4 and a p-value ≤ 0.05 in the cell layer of BM-MSC (n = 3) and lung-MSC (n = 3). Unsupervised hierarchical clustering reveals two protein clusters (64 proteins were found to be down-regulated and 288 proteins up-regulated in lung-MSC compared to BM-MSC). Data are presented as z-score, where green color codes for higher expression and red color codes for lower expression. LU, lung-derived MSC; BM, bone marrow-derived MSC.

**Figure 4:**
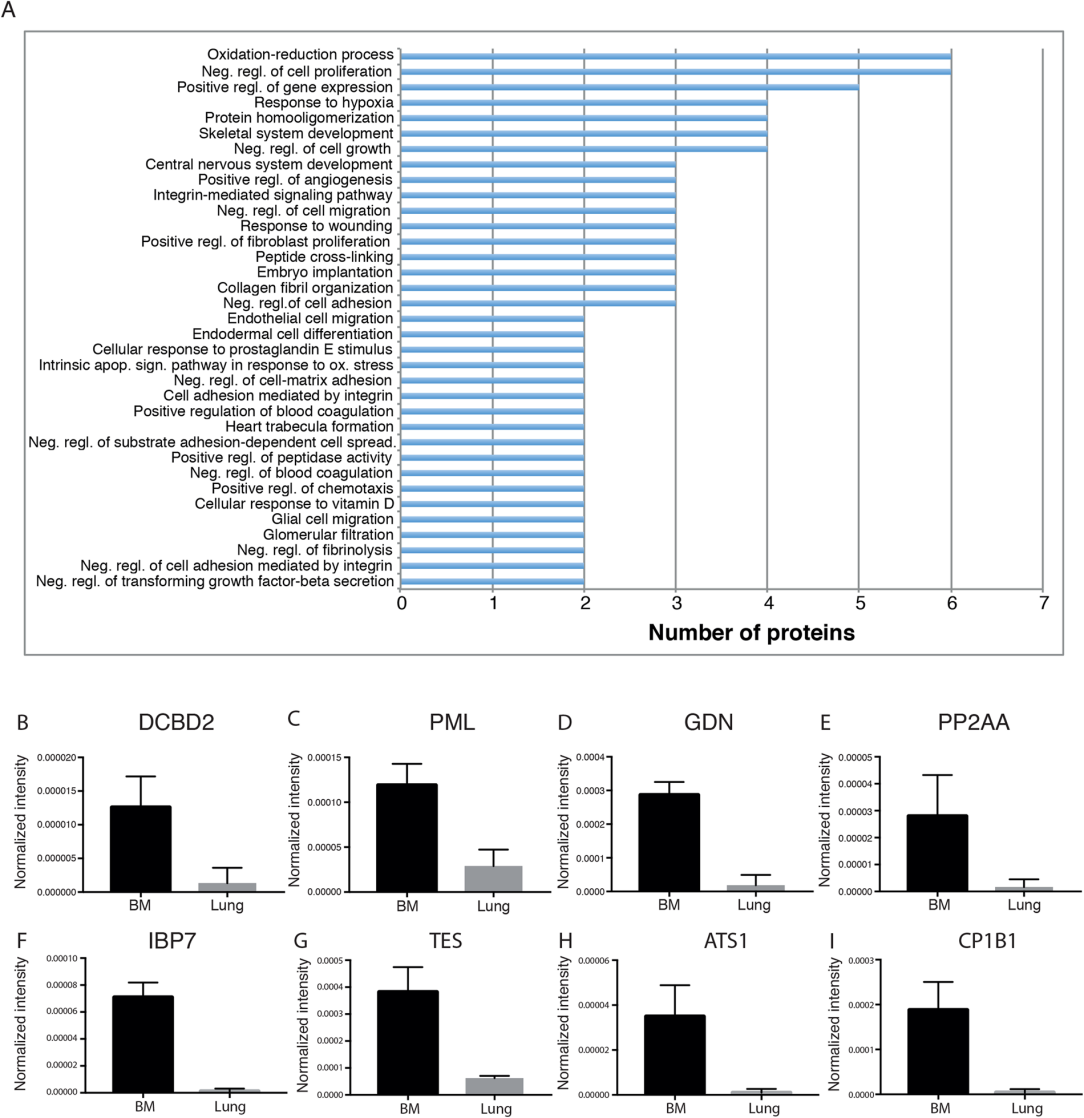
Enrichment analysis of biological processes using DAVID software. (**A**) Cellular compartment enrichment analysis of biological processes on proteins that were down regulated in cell layer of lung-MSC (n = 3) compared to bone marrow-MSC (n = 3) using the DAVID program. Processes with a FDR >10% containing more than two proteins are presented. The expression of proteins involved in negative regulation of cell growth (**B**,**C**,**D**,**E**) and negative regulation of proliferation (**C**,**D**,**F**,**G**,**H**,**I**) were measured using quantitative proteomic. Data are presented as normalized intensity and mean (±SD). Neg, negative; regl, regulation; apop, apoptosis; sign, signaling; ox, oxidative; DCBD2, Discoidin (CUB and LCCL domain-containing protein 2); PML, Promyelocytic leukemia protein; GDN, Glia-derived nexin; PP2AA, Serine/threonine-protein phosphatase 2A catalytic subunit alpha isoform; IBP7, Insulin-like growth factor-binding protein 7; TES, Testin; ATS1, A disintegrin and metalloproteinase with thrombospondin motifs 1 (ADAM-TS1); CP1B1, Cytochrome P450 1B1; BM, bone marrow-derived MSC; Lung, lung-derived MSC. The p-values were determined using RStudio with Benjamini and Hochberg correction for multiple testing.

The inclusion of MSC isolated from two different organs allowed us to study tissue-specific proteome changes in both cell layer and conditioned medium. As expected, this comparison revealed similarities between the two MSC populations, but importantly this comparison also revealed large differences in their proteomes. We found 288 proteins significantly enriched in lung-derived MSC cell layer (≥4 fold change, p ≤ 0.05) and 64 proteins that were significantly enriched in bone marrow-derived MSC cell layer (≥4 fold change, p ≤ 0.05) (Fig. 2b). Comparing the proteins secreted into the conditioned medium, we found 36 proteins that were significantly enriched in lung-MSC cultures (≥4 fold change, p ≤ 0.05), and 37 in bone marrow-MSC cultures (≥4 fold change, p ≤ 0.05) (Fig. 2c). Unsupervised hierarchical clustering of proteins identified in the cell layer clearly cluster into two distinct protein groups (Fig. 3). Two distinct protein clusters were also seen for the conditioned medium (Supplementary Figure 1). Analyzing the gene ontology (GO) terms associated with the proteins found to be significantly down-regulated in the cell layer of lung-derived MSC compared to bone marrow-derived MSC, we found 17 GO terms (FDR ≥ 10%, containing more than two proteins) including GO terms related to regulation of cell proliferation, and cell growth, response to wound healing, oxidation-reduction processes and positive regulation of gene expression (Fig. 4a). Based on previous findings we were especially interested in proteins involved in regulation of cell proliferation and cell growth^3^. Therefore, we extracted the proteins from the GO terms: negative regulation of cell proliferation and negative regulation of cell growth. We observed a significantly increased expression of discoidin (DCBD2, 9.5 fold change, p = 0.0192), promyelocytic leukemia protein (PML, 4.1 fold change, p = 0.0066), glia-derived nexin (GDN, 15.6 fold change, p = 0.0007), serine/threonine-protein phosphatase 2A catalytic subunit alpha isoform (PP2AA, 16.8 fold change, p = 0.0428), insulin-like growth factor-binding protein 7 (IBP7, 62.1 fold change, p = 0.0004), testin (TES, 6.1 fold change, p = 0.0040), a disintegrin and metalloproteinase with thrombospondin motifs 1 (ATS1, 35.9 fold change, p = 0.0138), and cytochrome P450 1B1 (CP1B1 or CYP1B1, 44.5 fold change, p = 0.0072) in bone marrow-derived MSC compared to lung-derived MSC (Fig. 4b–i). In addition, among the proteins that were found to be up-regulated in the cell layer of lung-derived MSC compared to bone marrow-derived MSC, we identified 52 GO terms (FDR ≥ 10%, containing more than two proteins). Among these terms, different biological processes such as positive regulation of NF-kappaB signaling, T cell receptor signaling pathway, regulation of mRNA stability, and response to drugs were identified (Supplementary Figure 2).

### Lung- and bone marrow-derived MSC are extracellular matrix producers

**Figure 5:**
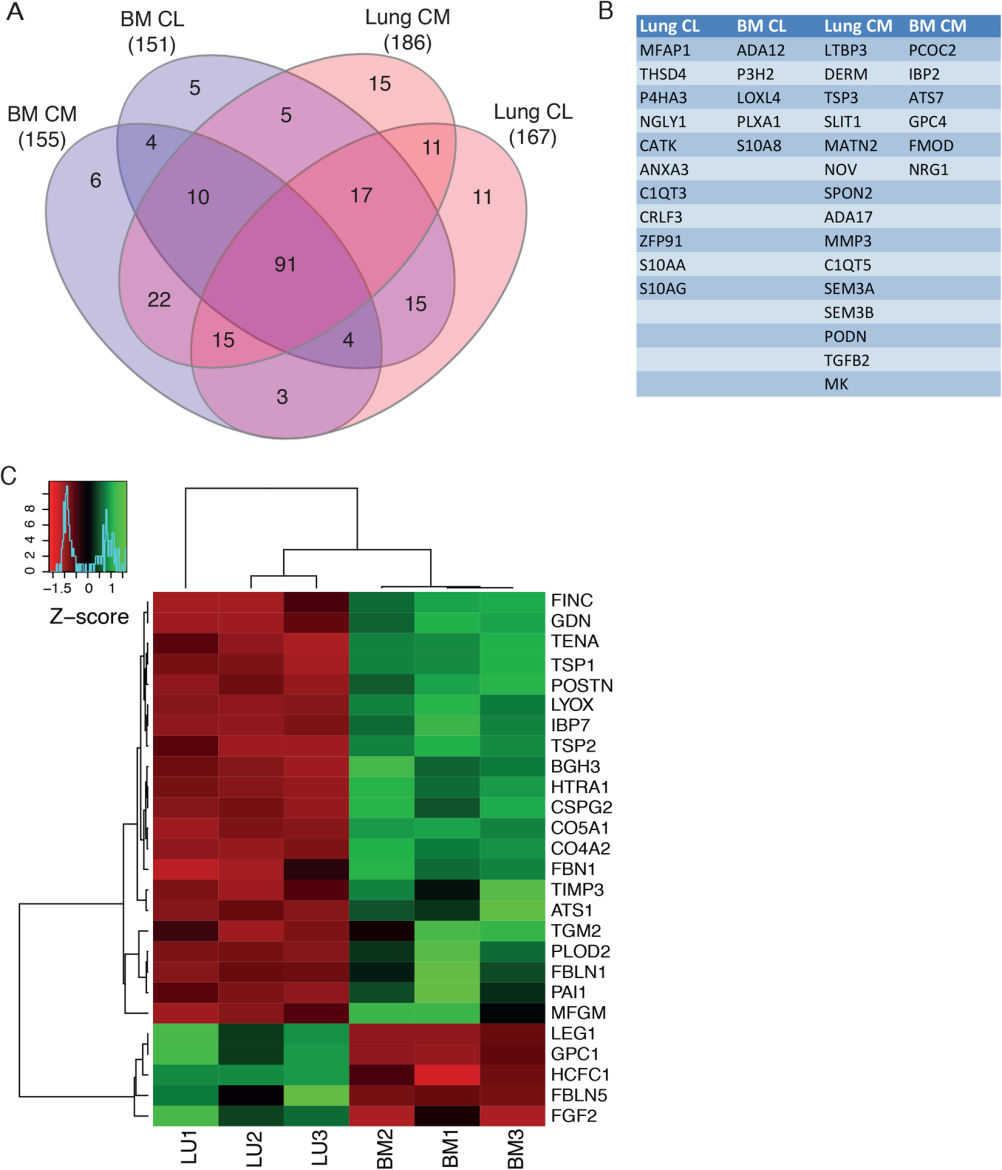
Label-free quantitative characterization of lung- and bone marrow-derived MSC matrisome (ECM) profiles using DIA-MS. (**A**) Venn diagram of the total number of extracellular matrix (matrisome) proteins identified by quantitative proteomics in each sample group (BM-MSC conditioned medium (n = 3), BM-MSC cell layer (n = 3), lung-MSC conditioned medium (n = 3) and lung-MSC cell layer (n = 3)). (**B**) A table of matrisome proteins only identified in one group of four. (**C**) A heat map of the significantly differentially expressed matrisome proteins with a fold change of ≥4 or ≤−4 found in the cell layer of BM-MSC (n = 3) and Lung-MSC (n = 3). Unsupervised hierarchical clustering reveals clear differences in matrisome profile between BM-MSC and lung-MSC. Data are presented as z-score, where green color codes for higher expression and red color codes for lower expression. BM, bone marrow-derived MSC; Lung, lung-derived MSC; CL, cell layer; CM, conditioned medium.

To study the ability of ECM production of lung- and bone marrow-derived MSC we used the *in silico* defined matrisome groups, described by Naba *et al*.^7^, to assign matrisome affiliation to our protein groups. We identified 167 ECM proteins in lung-derived MSC cell layer and 151 proteins in bone marrow-derived MSC cell layer (Fig. 5a). Among the identified ECM proteins, 11 proteins were exclusively identified in the lung-MSC cell layer samples, and 5 proteins were exclusively detected in the cell layer of bone marrow MSC (Fig. 5b). Fibronectin was one of the most abundant proteins detected in cell layer from lung- and bone marrow–derived MSC. However, in cell layer from bone marrow-MSC we detected significantly higher amount of fibronectin compared to lung-derived MSC (p = 0.0010). Furthermore, collagen I (collagen I AI p = 0.0034), and collagen VI (collagen VI A1 p = 0.0011, and collagen VI A2 p = 0.0027) were significantly higher expressed in cell layer from bone marrow-derived MSC. Interestingly, tenascin-C which is known to influence the number of hematopoietic stem cells in the bone marrow^9^ was significantly higher in bone marrow-MSC compared to lung-MSC (p = 0.0005). When we analyzed the ECM proteins secreted into the conditioned medium, we detected 186 proteins in lung-MSC cultures and 155 in bone marrow-MSC cultures (Fig. 5a). Among the identified ECM proteins, 15 proteins were exclusively identified in the conditioned medium collected from cultures with lung-derived MSC, and 6 proteins were exclusively detected in the conditioned medium of bone marrow-MSC (Fig. 5b). In the conditioned medium, fibronectin was the most abundant protein, but no significant difference between lung- and bone marrow-derived MSC was detected. Furthermore, high amounts of collagens I and VI was detected in the conditioned medium, but no significant difference between the two groups were detected. However, collagen III was significantly increased (p = 0.0294), and collagen V (p = 0.0085) was significantly decreased in the conditioned medium from lung-MSC compared to bone marrow-MSC. Unsupervised hierarchical clustering analysis of ECM proteins identified in the cell layer clearly clusters the analyzed samples in two distinct groups (Fig. 5c). Among all identified matrisome proteins 39% were detected in all four groups *i*.*e*. cell layer and conditioned medium from lung- and bone marrow-derived MSC. For cell layer and conditioned medium 66% and 68% were found in both lung-MSC and bone marrow-MSC samples, respectively. A detailed description of the matrisome composition identified in cell layer and conditioned medium of lung- and bone marrow-derived MSC are presented in Supplementary Figure 3.

### Increased CYP1B1 expression decreases the proliferation rate of lung-derived MSC

**Figure 6:**
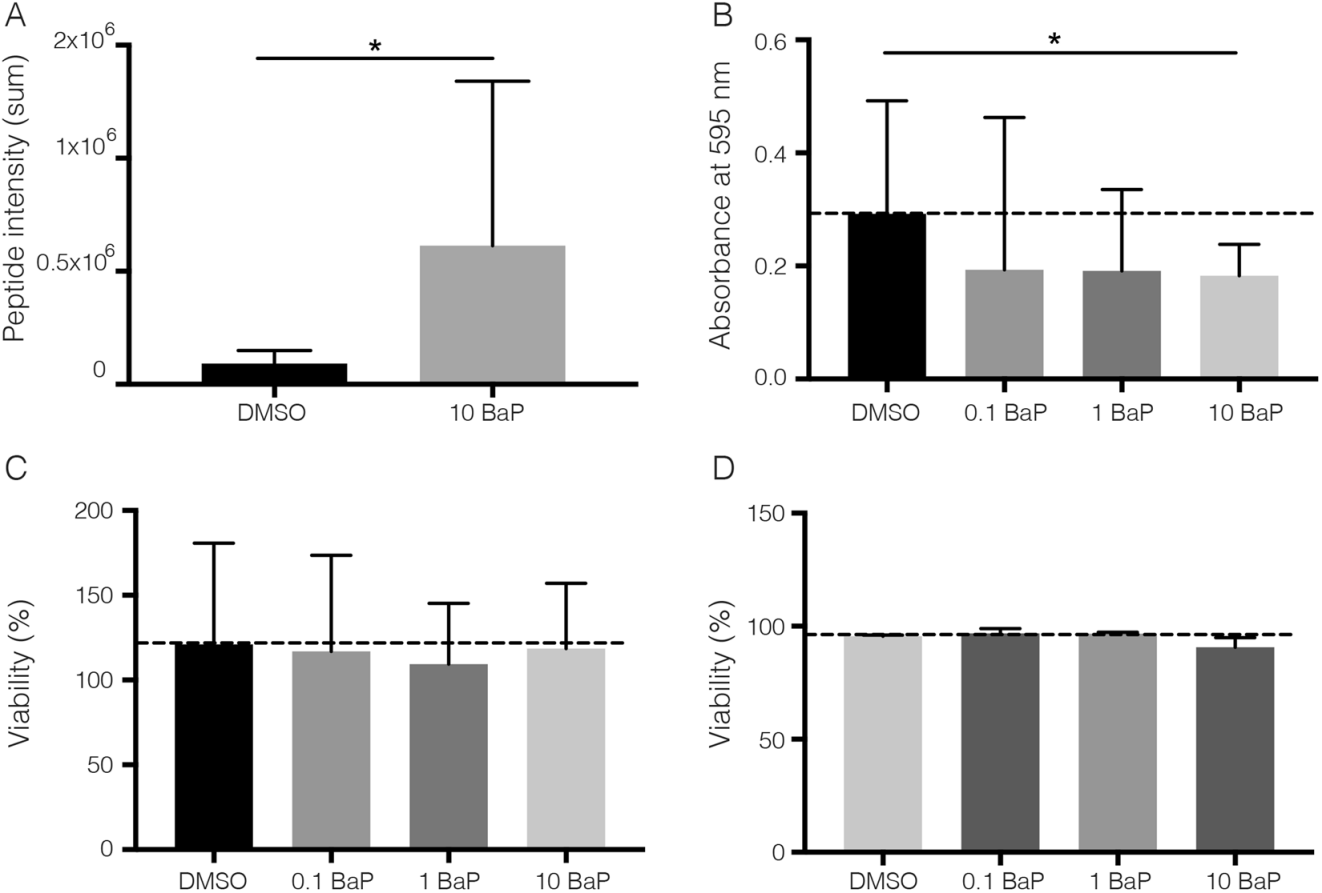
Verification of CYP1B1 by SRM-MS and functional evaluation of its effect on proliferation. (**A**) Selected reaction monitoring analysis of CYP1B1 expression before and after stimulating lung-derived MSC with 10 µM Benzo[a]pyrene (BaP) for 24 hours (n = 4). (**B**) Stimulating Lung-MSC with 10 µM BaP for 24 hours decreased the proliferation rate (n = 5). No significantly increase in cell death was observed when stimulating Lung-MSC with 10 µM BaP up to 48 hours according to LDH analysis (n = 4) (**C**) or Trypan blue staining’s (n = 2) (**D**). Data are presented as mean (±SD).

To further validate the proteomic data functionally, we evaluated whether proliferation rate of lung-derived MSC were affected by increased CYP1B1 expression, a protein known to have anti-proliferative properties. First, we assessed the CYP1B1 levels in lung-derived MSC with or without the stimulator Benzo[a]pyrene (BaP) using selected reaction monitoring (SRM) MS. Stimulating MSC with 10 µM BaP for 24 hours led to a significant increased CYP1B1 expression (summary of four peptides, p = 0.0286) on lung-derived MSC (Fig. 6a). To test if increased CYP1B1 expression decreased the proliferation rate of cultured MSC, we analyzed the proliferation rate of BaP-stimulated lung-derived MSC. Our data demonstrate that stimulating lung-MSC with 10 µM BaP for 24 hours significantly decreased the proliferation rate (Fig. 6b). Importantly, we did not observe a significant increase in cell death when stimulating MSC with 10 µM BaP up to 48 hours (Fig. 6c,d).

## Discussion

In this study, we performed an in-depth quantitative mass spectrometry based strategy with the aim to comprehensively characterize and compare the proteomes of MSC isolated from lung tissue and bone marrow aspirates. This is the first quantitative proteome characterization comparing the matrisome profile (ECM profile) of lung derived-MSC and the well-characterized bone marrow-derived MSC. Finally, we identified the enzyme CYP1B1 as a target protein of negative regulation of proliferation and cell growth of lung-derived MSC.

Until now, proteome characterizations of MSC have been performed without the ability to analyze the amount of the identified proteins expressed in the sample^10–12^. Therefore, we performed a quantitative characterization of the proteome of MSC. With this strategy we assess the tissue characteristic MSC proteome profiles to a depth of 3622 and 2772 proteins in cell layer and 974 and 961 proteins in the conditioned medium for lung-MSC and bone marrow-MSC, respectively. Surprisingly we identified a larger number of proteins in the cell layer of lung-MSC compared to bone marrow-MSC. We investigated the intensities of the identified proteins, and found that the number of high intense and low intense proteins were similar between lung- and bone marrow-derived MSC. It was instead the number of medium intense proteins that were increased in the lung-MSC cell layer samples. In the conditioned medium, we were able to identify equal number of proteins from lung- and bone marrow-derived MSC (Supplementary Figure 4). Recently, Anderson *et al*. published a comprehensive proteomic study on bone marrow-derived MSC and exosomes isolated from MSC cultures. In this study the authors identified 6342 proteins from bone marrow-MSC cell pellets and 1927 proteins in MSC-derived exosomes. This proteome depth was achieved by fractionation of the cell pellets. However, these results are comparable with the depth (6337 proteins were detected) that we achieved from SDS-PAGE fractionation of MSC samples^12^.

In accordance with previous reports on other cell types^13, 14^, our data demonstrate that MSC isolated from different tissues share many similarities, but importantly unique tissue-specific differences within their proteome profile. Geiger *et al*. have previously published a study where they compared the proteome of 11 common cell lines. In this study, they found that most cell lines expressed proteins that clustered with the original function of the cell type. For example, Jurkat cells expressed proteins that were enriched for the gene ontology terms positive regulation of lymphocyte activation and immune system process^13^. Interestingly, our data show that the same cell type, *i*.*e*. MSC, isolated from different tissues expressed different proteins. However, Geiger *et al*. also demonstrated that despite the cell type specific pattern most of the proteome proteins were similar between the different cell lines^13^. Lundberg *et al*. reported that 65% of the identified proteins were similar between the three evaluated cell lines^14^. These publications, together with our results, indicate that a large part of the cell proteome is similar in different cell types *i*.*e*. “house keeping proteome” or “core proteome”. However, when comparing the proteome of different cell types it is clear that cell-specific or tissue-specific proteins exists, suggesting that these proteins are important for the functional role of that particular cell type or tissue.

For a long time, the ECM was considered only as a structural component that supported and organized the tissue. ECM has recently been shown to exert multiple functions *e*.*g*. functioning as a reservoir for cytokines, growth factors and chemokines, as well as influencing cell proliferation, migration and differentiation^15^. For example, MSC cultured on standard tissue-culture polystyrene have an altered proliferation rate and morphology compared to MSC cultured on ECM produced by MSC^16^. Interestingly but not surprisingly, MSC have been demonstrated to play an important role in the bone marrow by providing a stem cell niche for the hematopoietic system, thus ensuring proper function of the hematopoietic system^9^ and therefore we hypothesized that lung-derived MSC produced ECM proteins may be important for the pulmonary stem cell niche/microenvironment. Utilizing matrisome filtration on the proteome data we were able to demonstrate that MSC are matrix producing cells, which secreted ECM proteins into the conditioned medium as well as deposited ECM in the pericellular niche *i*.*e*. cell layer. We were able to identify 235 ECM proteins and in accordance with Harvey *et al*. and Ragelle *et al*., our data demonstrate that fibronectin is one of the most abundant ECM protein produced by MSC^16, 17^. Fibronectin is a large glycoprotein with a variety of cellular properties like cell adhesion and migration^18^. It has been demonstrated that adhesion of fibronectin to MSC regulates the migration of MSC during vascular remodeling by induction of PDGFR-beta signaling^19^. In accordance with what have previously been reported by Siebertz *et al*. glypican was detected in the bone marrow samples^20^. Glypican is a heparan sulfate proteoglycan. Interestingly, several studies have suggested that heparan sulfate proteoglycans are important components of the microenvironment of hematopoietic tissues and might play an important role in the interaction of stem and stromal cells^20–22^. Our results demonstrate that lung-derived MSC express significantly higher amount of glypican I in the cell layer compared to bone marrow-MSC (p = 0.0034). Another important component of the microenvironment that might play an important role is tenascin-C. Tenascin-C is a glycoprotein known as a critical component in the bone marrow, which is required for hematopoietic regeneration^9, 23^. Tenascin-C was found to be expressed both by lung- and bone marrow derived MSC, but the amount was significantly higher in cell layer produced by bone marrow-derived MSC. Furthermore, we were able to identify tissue-specific patterns within the ECM profiles, reflected by 37 ECM proteins that were only expressed by lung-MSC and 15 ECM proteins only by bone marrow-MSC. Among the lung-specific ECM proteins hepatocyte growth factor (HGF) was detected. HGF is essential for organ development during fetal embryogenesis, and endogenous HGF have been reported to be necessary for self-repair of injured organs such as liver and lung^24^.

Next, we performed an enrichment analysis on the significantly differentially expressed proteins in the cell layer samples between lung- and bone marrow-derived MSC. Our results demonstrate higher expression of proteins involved in biological processes such as negative regulation of proliferation and cell growth in cell layers isolated from bone marrow-derived MSC. These results are in accordance with what we have previously reported *i*.*e*. that bone marrow-derived MSC have a lower proliferation rate compared to lung-derived MSC^3^. One of the identified proteins was CYP1B1 which is an enzyme, conserved across several species in the early embryo during the development, that utilizes endogenous substrates to generate intracellular messengers. CYP1B1 is expressed by different cell types such as mesenchymal cells, stromal cells and extrahepatic epithelial^25^. Our data demonstrate that bone marrow-derived MSC express higher levels of CYP1B1 compared to lung-derived MSC. The high levels of CYP1B1 expressed by bone marrow-derived MSC have been described by Heidel *et al*. to be important for the metabolism of the immunosuppressor 7,12-dimethylbenza[a]anthracene (DMBA) known to induce apoptosis of pre-B cells^26^. Palenski *et al*. reported that pericytes isolated from mouse retinas expressed CYP1B1. Furthermore, they demonstrated that lack of CYP1B1 was associated with decreased cell death and enhanced proliferation rate. Importantly, they reported that the lack of CYP1B1 was associated with sustained NF-kappaB activation and increased oxidative stress^25^. In accordance with Palenski *et al*., our enrichment analysis show that lung-MSC, expressing lower levels of CYP1B1, have a significantly higher expression of proteins involved in the biological process “positive regulation of l-kappaB kinase/NF-kappaB signaling” (Supplementary Figure 2). However, as we have not investigated other possible pathways that might regulate proliferation rate, we cannot draw any definitive conclusions.

Taken together, this study demonstrates significant differences in the proteome of MSC isolated from lung tissue compared to and bone marrow-derived MSC. Furthermore, we describe the matrisome profile of lung-derived MSC for the first time, which displayed significant differences compared to the well-characterized bone marrow-derived MSC. It is likely that the MSC spectral library described here can further advance our understanding regarding the physiological role of endogenous lung MSC.

## Material and Methods

### Cell culture

Lung-MSC were isolated from transbronchial biopsies (parenchymal tissue, biopsies were taken at Skåne University Hospital, Lund, Sweden) from lung-transplanted patients as previously described^4^. Briefly, biopsies were washed, cut in to smaller pieces and further enzymatically dissociated using Dulbecco’s phosphate buffered saline (DPBS) containing collagenase type I (300 U/ml, Gibco BRL, Paisley, USA), hyaluronidase (1 mg/ml, Fisher Scientific) and DNAse (1 µl/ml, Qiagen, Solna, Sweden). Mononuclear cells (MNC) were seeded in StemMACS MSC expansion medium (MACS Miltenyi Biotec, Bergisch Gladbach, Germany) supplemented with 1% antibiotic antimycotic solution (Sigma Aldrich, Stockholm, Sweden). Lung function was measured by standard spirometry and only patients that did not have Bronchiolitis Obliterans Syndrome (according to the International Society for Heart and Lung Transplantation guidelines)^27^ *i*.*e*. normal lung function were included in this study. The regional ethical committee approved this study and all patients gave their written informed consent to participate in the study (Lund, Sweden 2005/560 and 2014/612). All experimental protocols were carried out in accordance with approved guidelines from the Swedish regional (Lund) ethical committee (application number: 2005/560 and 2014/612). No organs or tissues were procured from prisoners.

Bone marrow-MSC were isolated from healthy donors as previously described^3, 28^. Briefly, bone marrow aspirates from the iliac crest bone were collected and MNC were isolated by density-graded centrifugations and further seeded in StemMACS MSC expansion medium (MACS Miltenyi Biotec) supplemented with 1% antibiotic antimycotic solution (Sigma Aldrich). The Swedish regional (Lund) ethical committee approved this study and all patients gave their written informed consent to participate in the study (Lund, Sweden 2009/532), and all methods were carried out in accordance with approved guidelines (application number: 2009/532).

### Flow cytometry

Cultured MSC (passage 3–4) were harvested with 0.05% trypsin-EDTA, non-specific binding was blocked and the cells were stained with the following direct-conjugated antibodies CD31 (cat no. 555445, BD Pharmingen), CD34 (cat no. 555821, BD Pharmingen), CD45 (cat no. 345808, BD), CD73 (cat. no. 550257, BD Pharmingen), CD90 (cat. no. 559869, BD Pharmingen), CD105 (cat. no. 561443, BD Pharmingen), and CD146 (cat. no. 550315, BD Pharmingen) as described previously^4^. Corresponding isotype controls were all from Becton Dickinson. Dead cells were excluded using 7-amino-actinomycin D staining and doublets were excluded by gating on SSC-H versus SSC-W and FSH-H versus FSC-W. Samples were analyzed on a LSR Fortessa (BD Bioscience) using the BD FACS Diva software version 8.0.1 (BD Bioscience). Data analyses were performed using FlowJo software v.10.1 (Tree star, Ashland, Orego, USA).

### Mass spectrometry

#### Cell culture for mass spectrometry analysis

Cultured MSC (passage 3) from lung tissue and bone marrow aspirates were seeded into 6-well plates and cultured in StemMACS MSC expansion medium (MACS Miltenyi Biotec) supplemented with 1% antibiotic antimycotic solution (Sigma Aldrich) under standard culture conditions (37 °C and 5% CO_2_). When 80–90% confluence was achieved cells were washed with Dulbecco’s phosphate buffered saline (DPBS) and 2 ml DMEM (without serum and phenol red, Sigma Aldrich, cat no. D5921), supplemented with 1% glutamine were added. After 24 hours, conditioned medium was collected and cell layer was washed with DPBS. Conditioned medium and cell layer samples were stored at −20 °C. For SRM analysis, MSC (passage 4–5) were cultured until 80–90% confluence followed by stimulation of 10 µM Benzo[a]pyrene (BaP) (Sigma-Aldrich) in StemMACS MSC expansion medium supplemented with 1% antibiotic antimycotic solution for 24 hours.

#### Mass spectrometry sample preparation

To analyze proteins in the cell layer, cell culture plates were thawed and scraped. Cell layer samples were diluted in SDS-PAGE sample buffer (0.5 M Tris-HCl, 0.5 M glycerol, 10% SDS, 0.5% beta-mercaptoethanol, 0.5% bromphenol blue), boiled at 95 °C for 5 minutes at 800 rpm, followed by centrifugation at 14.000 × g for 10 minutes. To analyze proteins released into the conditioned medium, two ml medium was dried in a vacuum centrifuge and prepared using the in-solution protein digestion protocol as described previously^29^. Protein amounts were determined using Pierce BSA Protein Assay Kit (Thermo Scientific, cat. no. 23225) according to manufacturer’s instructions. For construction of the spectral library 50 µg of each cell layer (n = 2) respective conditioned medium (n = 2) were separated on SDS-PAGE (4–15% Criterion TGX gels, BioRad laboratories Inc., Hercules, CA, USA) for 15 min at 60 V followed by 45 min at 200 V, and the gel was stained with GelCode Blue Safe Protein Stain (Thermo Scientific, Rockford, IL, USA). Each lane was cut into 45 bands that were pooled into 10 total fractions and prepared for in-gel digestion as previously described^30^. DIA samples (lung MSC n = 3, and bone marrow MSC n = 3) were prepared as described above, with the following modifications: the samples were separated on SDS-PAGE for 10 minutes to allow the samples to enter the gel, followed by extraction of the entire lane as one MS injection. SRM samples (lung MSC n = 4) were scraped and diluted in NP-40 buffer (1% Flukta/Nonidet P40 substitute, 50 mM Tris, 150 mM Sodium Chloride and complete mini). To enrich and select for CYP1B1, 100 ug of one sample were SDS-PAGE separated into 45 bands, pooled and prepared as described above. The size of CYP1B1 was determined and only bands close to the CYP1B1 protein size were cut out and analyzed for all of the samples (100 ul/sample).

#### Mass spectrometry analysis

LC-MS/MS analysis was performed on a Q-Exactive Plus mass spectrometer (Thermo Fisher Scientific). Peptide separation was carried out by an EASY-nLC 1000 liquid chromatography system (Thermo Fisher Scientific) connected to an RP-HPLC EasySpray column (ID 75 µm × 25 cm C18 2 µm 100 Å resin (Thermo Fisher Scientific)). Solvent A (0.1% formic acid) and solvent B (0.1% formic acid, 100% acetonitrile) was used to run a linear gradient from 5% to 30% of solvent B. For cell layer samples a 120 min gradient was used whereas for the conditioned medium samples a 60 min gradient was used, both at a flow rate of 300 nl/min. The data acquisition mode was set to obtain one high resolution MS scan (70.000 at 200 m/z). The 15 most abundant ions from the preceding MS scan were fragmented by higher energy induced collision dissociation (HCD) and MS/MS fragment ion spectra were acquired at a resolution of 17.500 at 200 m/z. Charge state screening was enabled and unassigned or singly charged ions were rejected. The dynamic exclusion window was set to 40 for the 120 min gradient and to 20 for the 60 min gradient. Only MS precursors that exceeded a threshold of 1.7e4 were allowed to trigger MS/MS scans. The ion accumulation time was set to 100 ms (MS) and 60 ms (MS/MS) using an AGC target setting of 1e6 (MS and MS/MS). For DIA data the same acquisition setting was set as described previously^30^. Briefly, 32 consecutive 26 Da precursor isolation windows followed each MS scan. All peptides for CYP1B1 (Q16678) identified using Data Dependent Acquisition (DDA) were exported to the SRM library using Tramler^31^. The final SRM assay contained 82 transitions that consisted of 15 peptides that were acquired in a non-scheduled mode. The peptides were separated with a nonlinear gradient of 3% solvent B (0.1% formic acid, 100% acetonitrile) over 5 min, 3–15% solvent B over 3 min, 15–35% solvent B over 34 min, and 90% solvent B over 3 min. The SRM measurements were performed on a TSQ Quantiva triple quadruple mass spectrometer (Thermo Scientific) connected to an EASY-nLC II high-pressure liquid chromatography system (Thermo Scientific). The peptides were loaded on an EASY-Spray column (Thermo Scientific; ID 75 µm × 15 cm) at a constant pressure of 280 bars and separated at a flow rate of 300 nl/min.

#### Mass spectrometry data analysis

Shotgun MS raw files were processed using the Trans-Proteomic Pipeline (TPP, version 4.7)^32^. All protein database searches were performed using X!Tandem Jackhammer TPP (20130651) against the manually reviewed human UniProt FASTA database (version November 2015) complemented with reversed sequences for all entries. Cysteine carbamidometylation was set as fixed peptide modification and methionine oxidation and hydroxylation of proline were set as variable peptide modifications. A precursor ion mass tolerance of 20 ppm and a fragment ion mass error tolerance of 50 ppm were used. Peptide Prophet probabilities of target and decoy hits were used to calculate the false discovery rate (FDR) at protein, peptide and spectrum level and set to 1% using the software tool Franklin^31^. Spectra of proteotypic peptides were summed to spectral counts. The same shotgun data was further used to build the MSC spectral library according to the Fraggle-Tramler-Franklin workflow as previously described^31^. Briefly, Fraggle was used to generate assays from shotgun MS spectra and combine them into spectral libraries. The selection of assays and generation of decoys were performed by Tramler. Franklin was used for multilevel FDR calculations on protein, peptide and assay level and set to 1%. The spectral library included 7749 proteins, 69755 peptides and 80987 assays. The spectra library data from this publication have been deposited to the ProteomeXchange Consortium via the PRIDE^33^ partner repository with the dataset identifier PXD006419. DIA files were analyzed using the software tool DIANA^34^. Protein quantification from DIA data were calculated by summing the intensities of their associated peptides. Protein abundances were normalized by dividing their intensities by the sum of all protein intensities per sample.

By using the *in silico* defined matrisome groups described by Naba *et al*.^7^ we assigned matrisome affiliation to our DIA quantified protein groups. The DIA-MS data from this publication have been deposited to the PeptideAtlas and are publicly available (see http://www.peptideatlas.org/PASS/PASS01017). SRM files were analyzed using Skyline (Skyline 3.6.0.10162 Mac Cross Lab) and a 1% FDR cutoff, a 0.1 minute peak min width, and a null distribution size of 500 was used. All acquired peptides were manually inspected in Skyline before summarizing, and four peptides were used for quantifications. The SRM raw data files were converted to numpressed mzML^35, 36^ using MsConvert in ProteoWizard 3.0.5930^37^. The SRM-MS data from this publication have been deposited to the PeptideAtlas and are publicly available (see http://www.peptideatlas.org/PASS/PASS01016).

### Proliferation

Cell proliferation was determined as previously described^38^. Briefly, lung-derived MSC (passage 3–6) were seeded in 96-well plates for 6 hours and then stimulated with StemMACS MSC expansion medium (MACS Miltenyi Biotec) containing 0.1 µM, 1 µM, or 10 µM Benzo[a]pyrene (BaP) (Sigma-Aldrich) for 24 and 48 hours. StemMACS MSC expansion medium containing Dimethyl Sulfoxide (DMSO) was used as control. Cells were fixed with 1% glutaraldehyde, stained with Crystal Violet dye and absorbance was measured at 595 nm utilizing a spectrophotometer plate reader. Proliferation rate was defined as absorbance at 24 hours or 48 hours subtracted by the absorbance after 6 hours.

### Cytotoxic analysis

#### Trypan blue dye exclusion assay

The cytotoxic effect of the CYP1B1 stimulator BaP was assessed manually by trypan blue dye exclusion assay. MSC (passage 3–6) were incubated for 48 hours in the presence of 0.1 µM, 1 µM, or 10 µM Benzo[a]pyrene (Sigma-Aldrich). Trypan blue (Sigma Aldrich) was added to the wells and the numbers of living (non-stained) and dead (blue stained) cells were calculated.

#### Lactate dehydrogenase assay

In addition, lactate dehydrogenase (LDH) assay was performed according to manufacturer’s instructions. Briefly, conditioned medium from the proliferation experiments were collected after 24 and 48 hours of stimulation (0.1 µM, 1 µM, or 10 µM BaP). LDH activity was measured utilizing an LDH detection kit (Roche, Germany, cat. no. 11644793001) and absorbance was measured at 490 nm using a spectrophotometer plate reader. Normal growth medium without cells served as background control, conditioned medium without BaP stimulation served as low control, and conditioned medium from cells stimulated with triton-X served as high control.

### Statistical analysis

Data were analyzed statistically using RStudio (version 0.99.903). All p-values presented in this study were Benjamini and Hochberg corrected for multiple testing. P-values ≤ 0.05 were considered as significant. Proteins involved in negative regulations of cell growth and proliferation were presented as mean (±SD). The SRM data were analyzed statistically using GraphPad Prism software (Version 7.0a) and data was presented as mean (±SD).

## Electronic supplementary material

Supplementary file
