# Supplementary material for: Quantitative proteomic characterization of lung-MSC and bone marrow-MSC using DIA-mass spectrometry
Source: Sci Rep. 2017 Aug 24;7:9316. doi: 10.1038/s41598-017-09127-y (PMC5570998; doi:10.1038/s41598-017-09127-y)
Supplement: Supplementary file 1 — Supplementary file [file 41598_2017_9127_MOESM1_ESM.pdf]

Title: Quantitative proteomic characterization of lung-MSC and bone marrow-MSC using DIA-mass spectrometry

Sara Rolandsson Enes[1]*, Emma Åhrman[1, 2], Anitha Palani[3], Oskar Hallgren[1], Leif Bjermer[4], Anders Malmström[3], Stefan Scheding[5, 6], Johan Malmström[2], Gunilla Westergren-Thorsson[1]

[1]Department of Experimental Medical Science, Lung Biology Unit, Lund University, 22184, Lund, Sweden
[2]Department of Clinical Sciences Lund, Division of Infection Medicine, Lund University,22184, Lund, Sweden
[3]Department of Experimental Medical Science, Matrix Biology, Lund University, 22184, Lund, Sweden
[4]Department of Respiratory Medicine and Allergology, Lund University and Skåne University Hospital, 22184, Lund, Sweden
[5]Lund Stem Cell Center, Lund University, 22184, Lund, Sweden
[6]Department of Hematology, Skåne University Hospital, 22184, Lund, Sweden

* Corresponding author: Sara Rolandsson Enes; sara.rolandsson_enes@med.lu.se

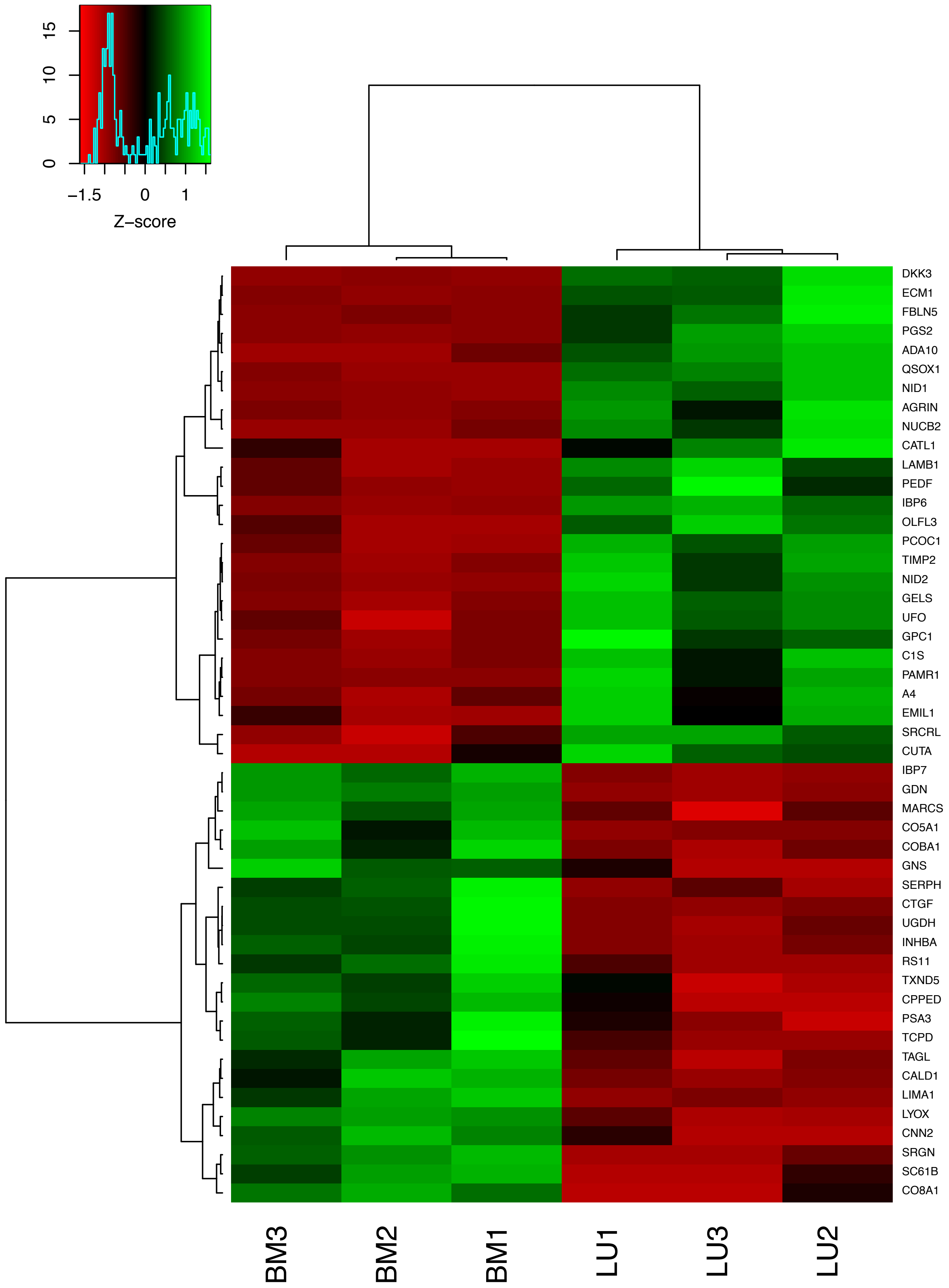

Supplementary Figure 1. A heat map of the significantly differentially expressed proteins with a fold change of ≥ 4 or ≤ -4 found in the conditioned medium of BM-MSC (n=3) and Lung-MSC (n=3). Unsupervised hierarchical clustering reveals two distinct proteins groups that differ between BM-MSC and Lung-MSC. Data are presented as z-score, were green color codes for higher expression and red color codes for lower expression.

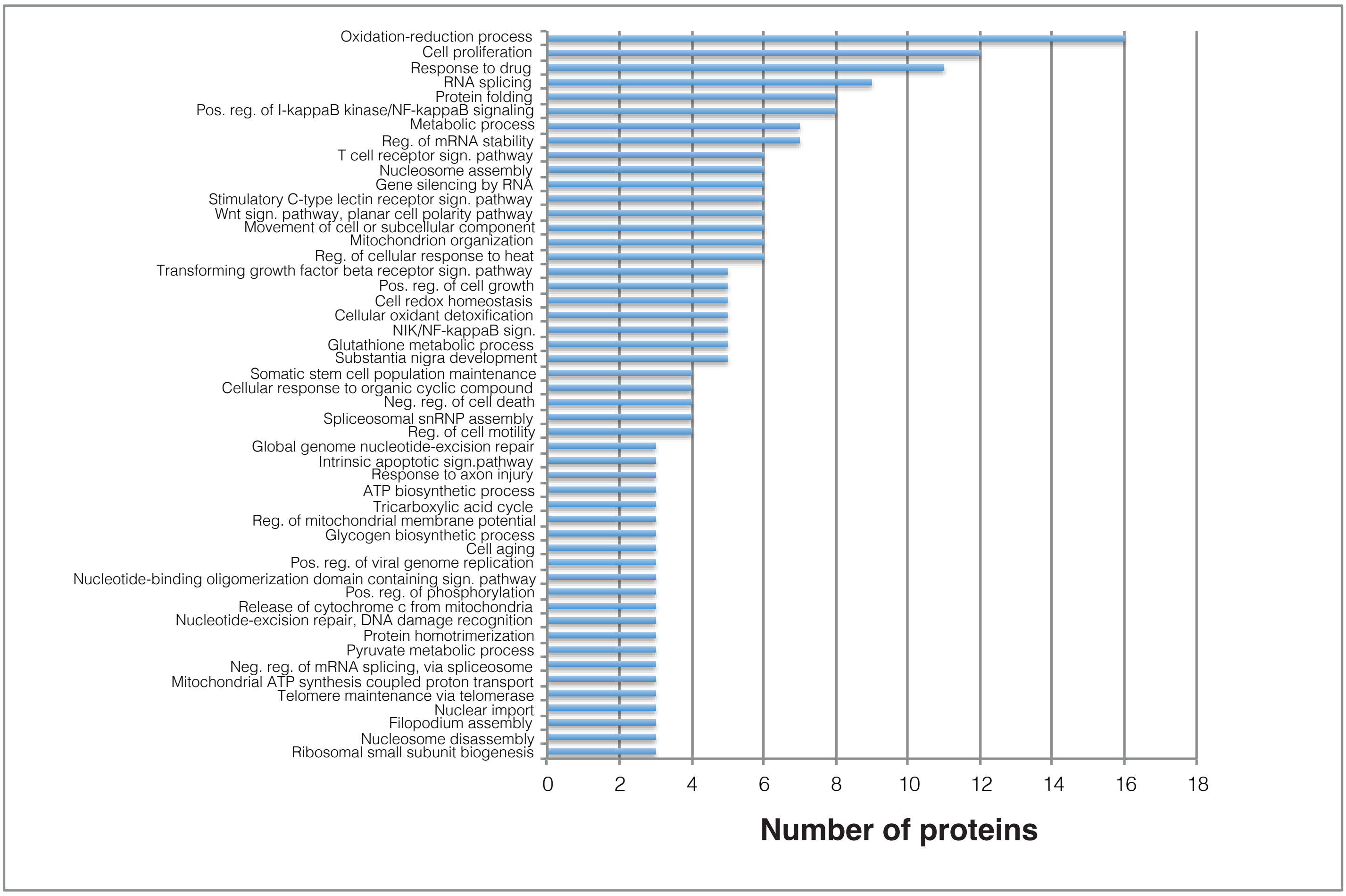

Supplementary Figure 2. Cellular compartment enrichment analysis of biological processes on proteins that were up regulated in cell layer of Lung-MSC (n=3) compared to BM-MSC (n=3) using the DAVID program. Processes with a FDR>10% containing more than two proteins are presented. Data are presented as normalized intensity and mean (± SD). Neg., negative; reg., regulation; pos., positive; sign., signaling

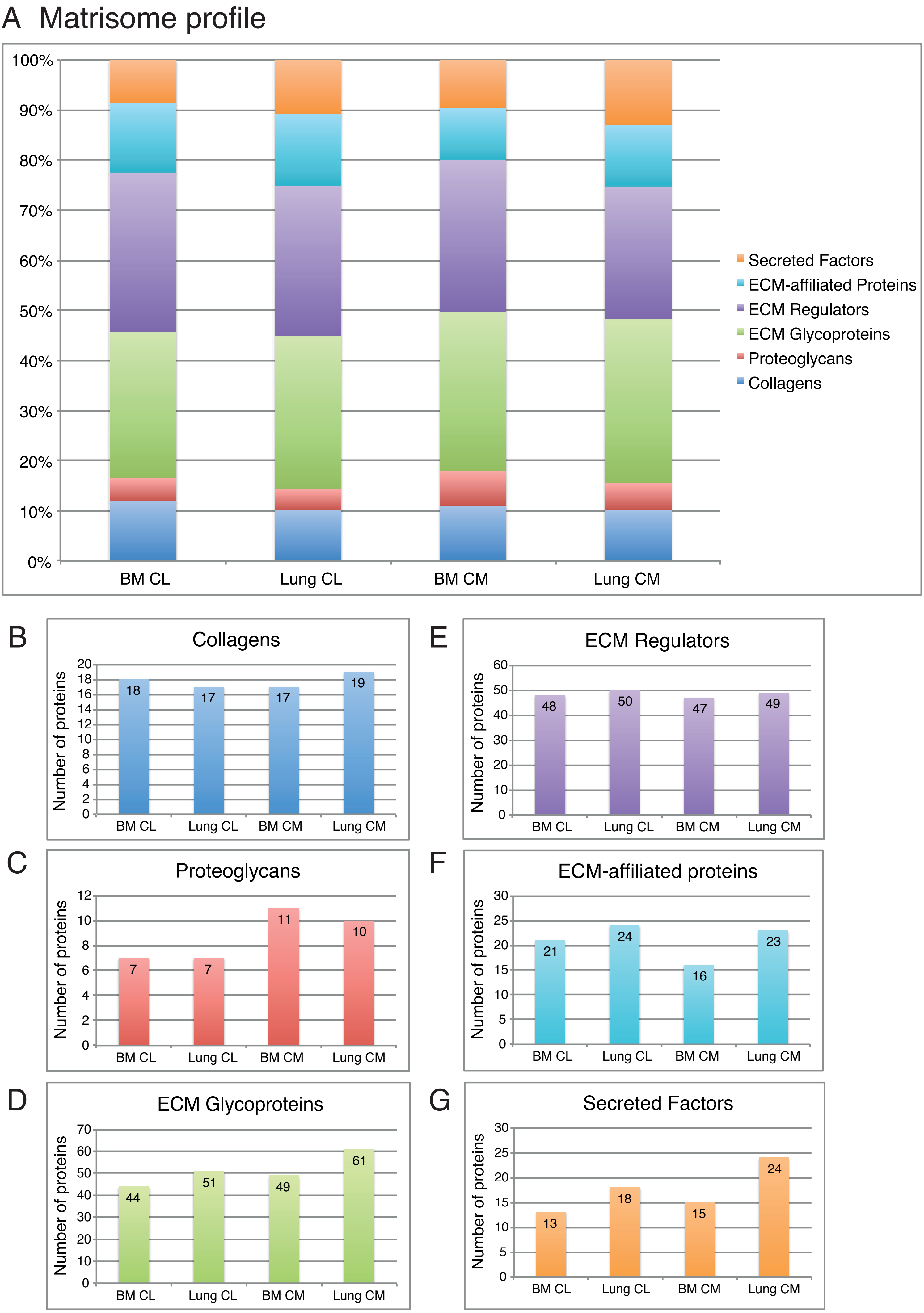

A Matrisome profile
100%
90%
80%
70%
60%
50%
40%
30%
20%
10%
0%
BM CL
Lung CL
BM CM
Lung CM
Secreted Factors
ECM-affiliated Proteins
ECM Regulators
ECM Glycoproteins
Proteoglycans
Collagens
B
Collagens
Number of proteins
18
17
17
19
C
Proteoglycans
7
7
11
10
D
ECM Glycoproteins
44
51
49
61
E
ECM Regulators
48
50
47
49
F
ECM-affiliated proteins
21
24
16
23
G
Secreted Factors
13
18
15
24

Supplementary Figure 3. (A) Total number of matrisome protein identified by quantitative proteomics in each sample group (BM cell layer (n=3), BM conditioned medium (n=3), lung cell layer (n=3), lung conditioned medium (n=3)). Total number of collagens (B), proteoglycans (C), glycoproteins (D), regulators (E), affiliated proteins (F) and secreted factors (G) identified by quantitative proteomics in each sample group. BM, bone marrow-derived MSC; Lung, lung-derived MSC; CL, cell layer; CM, conditioned medium.

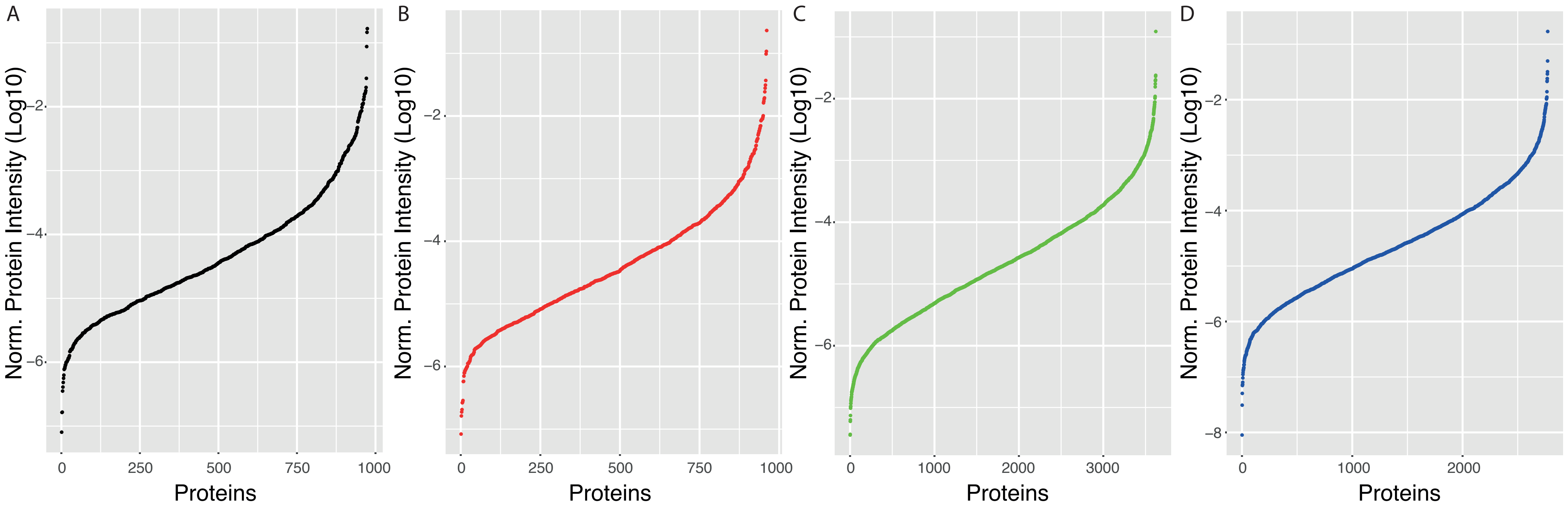

Supplementary Figure 4. Protein rank of proteins over their normalized protein intensity for (A) conditioned medium of Lung-MSC, (B) conditioned medium of BM-MSC, (C) cell layer of Lung-MSC, and (D) cell layer of BM-MSC. BM, bone marrow-derived MSC; Lung, lung- derived MSC.

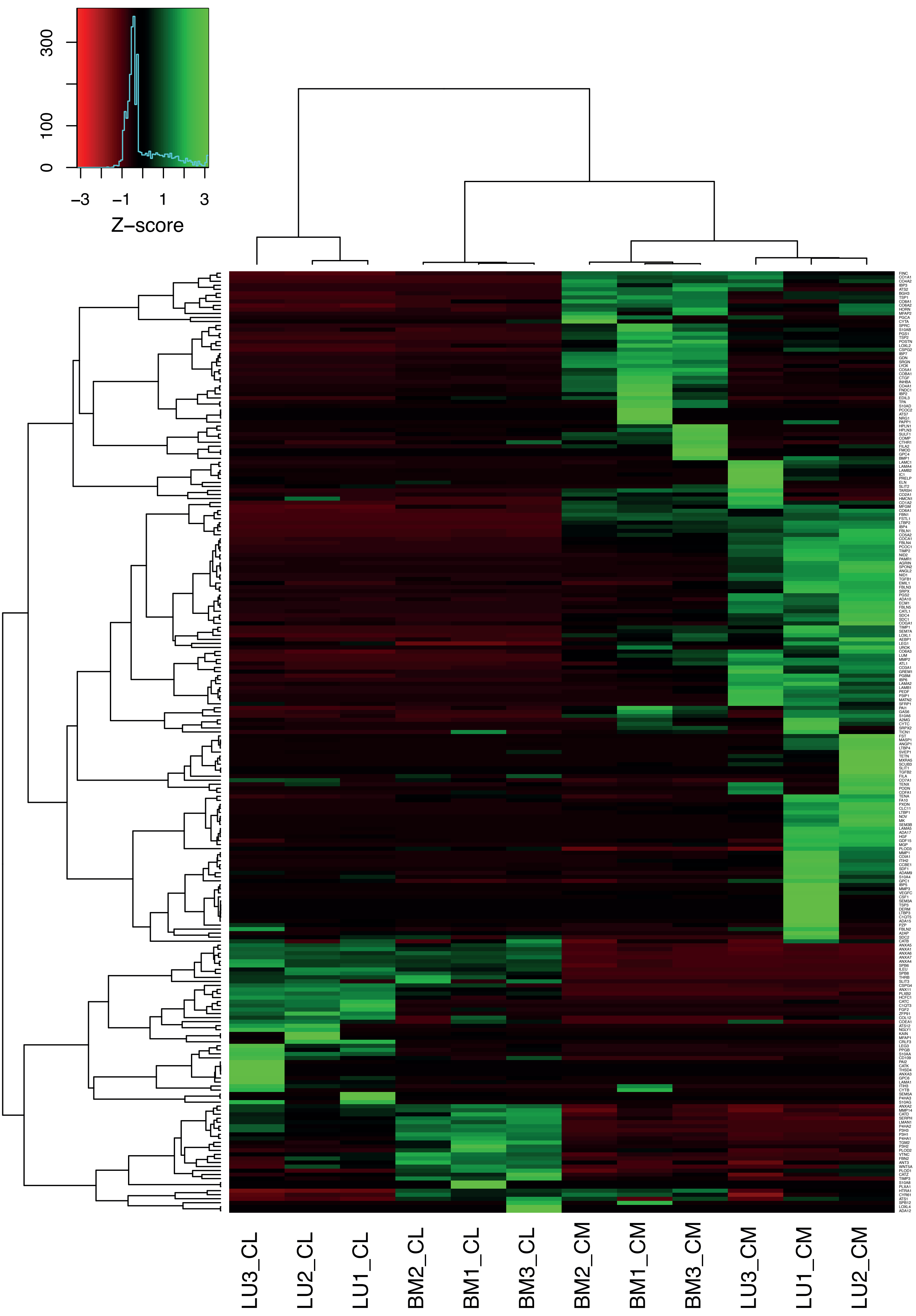

300
100
0
−3
−1
1
3
Z−score
LU3_CL
LU2_CL
LU1_CL
BM2_CL
BM1_CL
BM3_CL
BM2_CM
BM1_CM
BM3_CM
LU3_CM
LU1_CM
LU2_CM

Supplementary Figure 5. A heat map of all identified extracellular matrix proteins (matrisome proteins) identified in conditioned medium and cell layer of BM-MSC (n=3, n=3) and Lung- MSC (n=3, n=3). Unsupervised hierarchical clustering reveals clear differences between matrisome proteins between cell layer and conditioned medium as well as differences between lung- and bone marrow-derived MSC. Data are presented as z-score, were green color codes for higher expression and red color codes for lower expression. BM, bone marrow-derived MSC; Lung, lung-derived MSC; CL, cell layer; CM, conditioned medium.

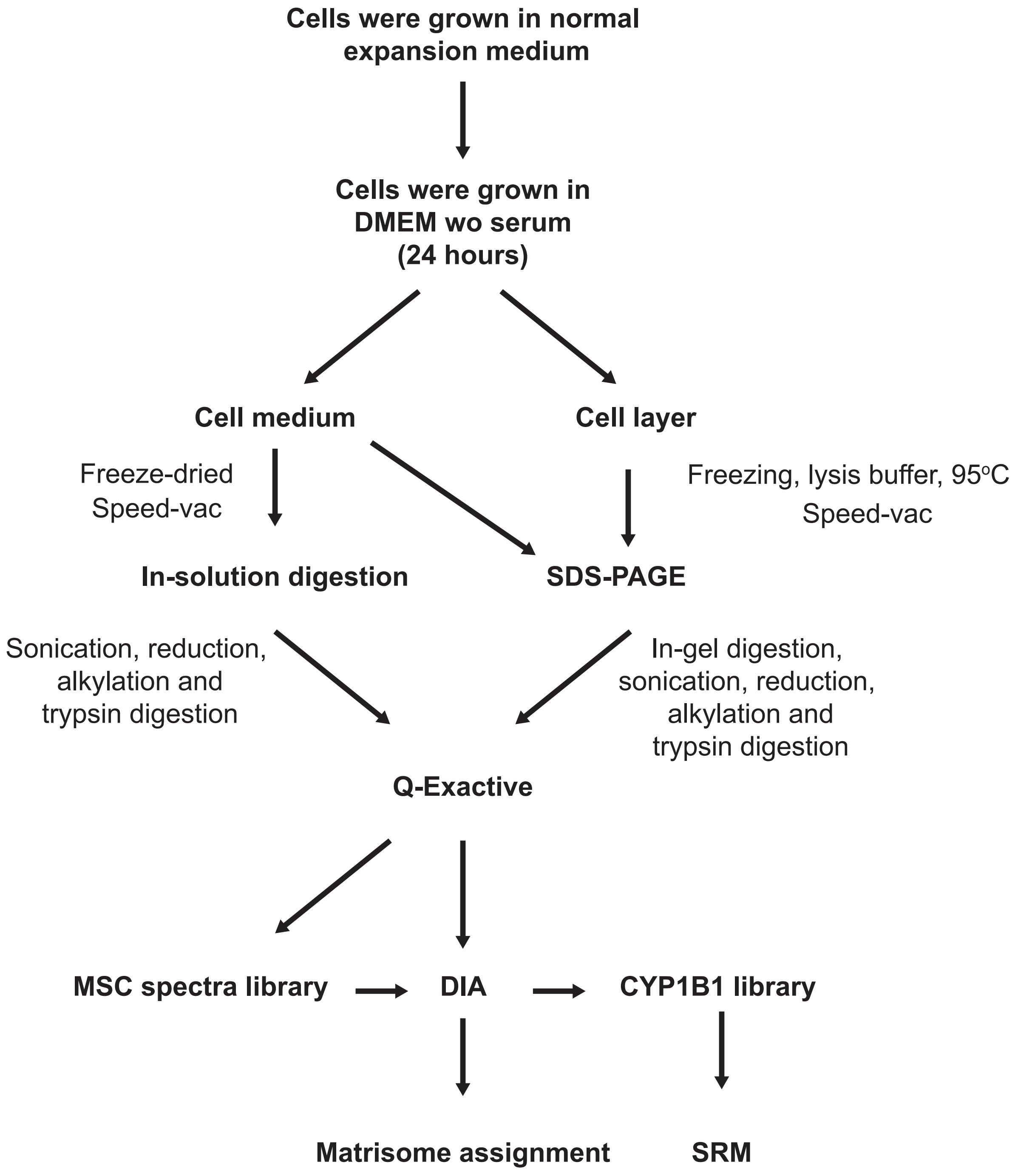

Supplementary Figure 6. Schematic picture of experimental work flow.
